# Synthetic lethal combinations of low-toxicity drugs for breast cancer identified *in silico* by genetic screens in yeast

**DOI:** 10.18632/oncotarget.26372

**Published:** 2018-11-20

**Authors:** Maximilian Marhold, Erwin Tomasich, Michael Schwarz, Simon Udovica, Andreas Heinzel, Paul Mayer, Peter Horak, Paul Perco, Michael Krainer

**Affiliations:** ^1^ Department for Internal Medicine I–Oncology, Comprehensive Cancer Center, Medical University of Vienna, Vienna, Austria; ^2^ Clinic of Internal Medicine I, Wilhelminenspital Wien, Vienna, Austria; ^3^ Emergentec biodevelopment, Vienna, Austria; ^4^ Department for Translational Oncology, German Cancer Research Institute (DKFZ), Heidelberg, Germany

**Keywords:** synthetic lethality, breast cancer, drug combination, cancer, treatment

## Abstract

In recent years, the concept of synthetic lethality, describing a cellular state where loss of two genes leads to a non-viable phenotype while loss of one gene can be compensated, has emerged as a novel strategy for cancer therapy. Various compounds targeting synthetic lethal pathways are either under clinical investigation or are already routinely used in multiple cancer entities such as breast cancer. Most of them target the well-described synthetic lethal interplay between PARP1 and BRCA1/2. In our study, we investigated, using an *in silico* methodological approach, clinically utilized drug combinations for breast cancer treatment, by correlating their known molecular targets with known homologous interaction partners that cause synthetic lethality in yeast. Further, by creating a machine-learning algorithm, we were able to suggest novel synthetic lethal drug combinations of low-toxicity drugs in breast cancer and showed their negative effects on cancer cell viability *in vitro*. Our findings foster the understanding of evolutionarily conserved synthetic lethality in breast cancer cells and might lead to new drug combinations with favorable toxicity profile in this entity.

## INTRODUCTION

The concept of synthetic lethality describes a relationship between two genes, wherein loss of one gene can be compensated, but simultaneous loss-of-function of both genes results in a non-viable phenotype [1]. For synthetic lethal interactions identified in S. cerevisiae, C. elegans or Drosophila spp., potential conservation in the human genome was suggested and further proposed as suitable targets for precision cancer therapy [2–4].

The first predicted human synthetic lethality gene interactions that led to the development of approved therapeutics were those of Breast Cancer genes 1 and 2 (BRCA 1/2) and poly (ADP–ribose) polymerase 1 (PARP1). Tumor cells deficient in either BRCA gene were highly susceptible to PARP inhibition, whilst this was not the case for BRCA wildtype cells [5, 6]. Whether by inhibiting PARP1-supported single-strand repair (SSR) or by trapping PARP at the DNA damage site, PARP inhibitors induce DNA lesions that require homology directed repair (HDR) [7–9]. Since both BRCA genes play essential roles in HDR pathways in humans [8], loss-of-function of either BRCA1 or 2 sensitizes cells to PARP inhibitors.

During recent years, successful efforts have been undertaken to discover further, novel and clinically significant synthetic lethal gene combinations, by using molecular biology approaches such as genetic RNA interference or CRISPR/Cas libraries [10–12]. Furthermore, *in silico* approaches using machine learning and network properties were shown to be valuable tools in identifying novel genetic as well as chemico-genetic interactions causing synthetic lethality [13, 14].

In this study, we used known negative genetic interactions in yeast to create a machine learning-based synthetic lethality predictor for human cancer cells. Based on novel synergies predicted *in silico* by our model, we were then able to verify the efficacy of the corresponding low-toxicity treatment combinations for breast cancer *in vitro*.

## RESULTS

### Creation of a systematic database of drug combinations in cancer therapy

Exploring the website “http://clinicaltrials.gov” for “cancer” and limiting results for trials in phase III or IV created a set of 6,665 trials, of which 643 met our requirements of oncologic indication and pharmacological intervention. These trials contained 790 different drug combinations. An additional 121 combinations were found in clinical practice. Taken together, a total of 911 different drug combinations spanning 119 indications were found (Supplementary Figure 2). 150 individual drugs were identified, targeting 285 different genes. The excess of targets compared to drugs was due to certain drugs targeting multiple proteins. Most frequently occurring drugs were cyclophosphamide, etoposide, and doxorubicin. Summarized in classes, half of the drugs were found to be antimetabolites, alkylating agents, antimicrotubule agents or anthracyclines (data not shown).

**Figure 1:**
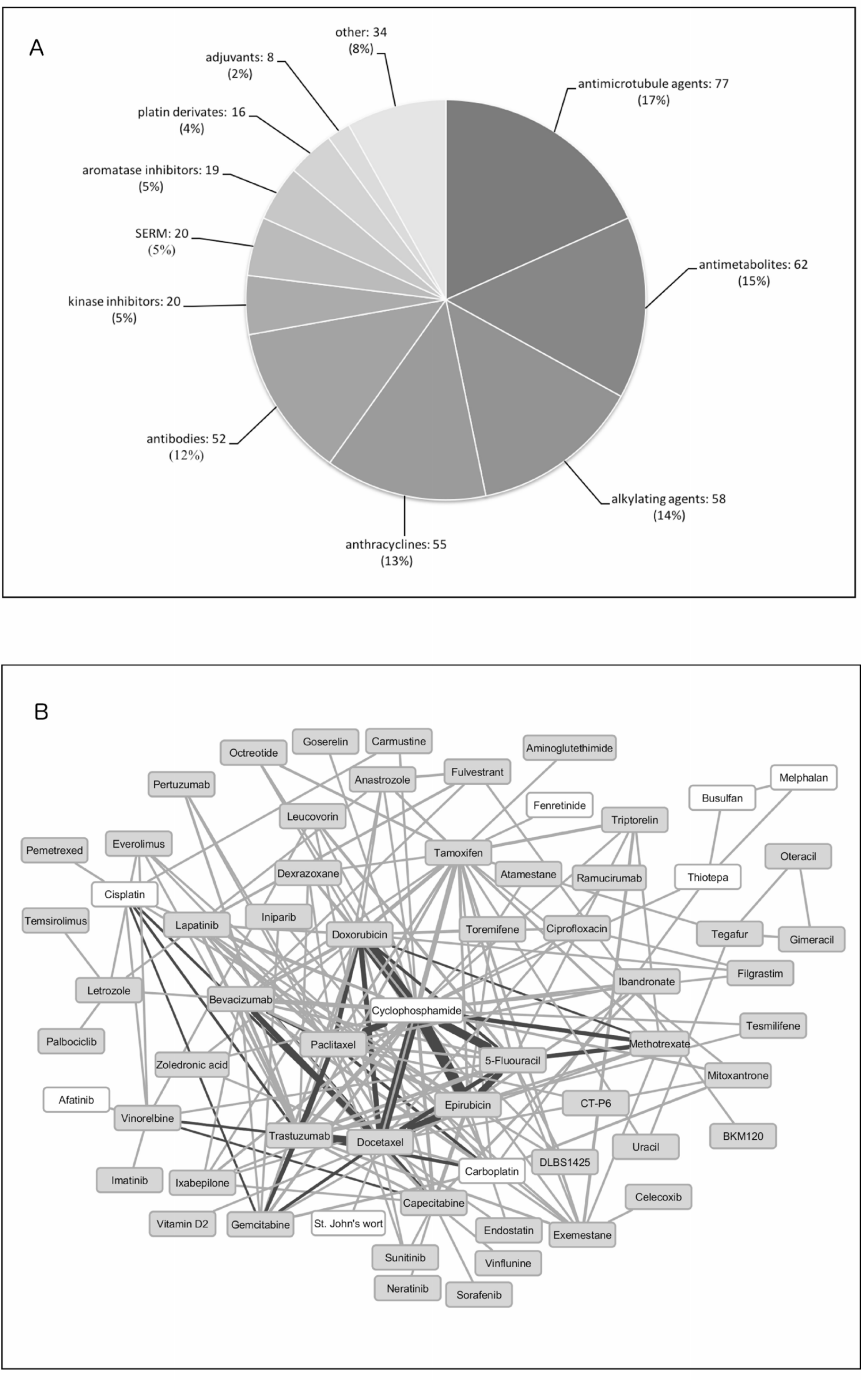
Drugs found in clinical practice and clinical trials for breast cancer (**A**) Pie chart representing drugs found in clinical practice for breast cancer clustered according to their mechanism of action. (**B**) Drug-drug combinations extracted from phase III and IV breast cancer clinical trials. White background drugs have no specific target protein. Dark lines represent drug combinations also used in clinical practice. Line width correlates with frequency in which drugs are combined.

Among the 119 different oncology treatment indications, breast cancer was the most frequent. As expected, this indication included the most drug combinations as well as the highest number of individual drugs (Table 1). The drug classes most abundant for breast cancer were antimicrotubule agents, antimetabolites, alkylating agents and anthracyclines (Figure 1A). A map showing drug combinations in breast cancer clinical trials was created and is illustrated in Figure 1B.

**Table 1:** Oncologic entities and assigned drug combinations

Disease term	Drug combinations	Individual drugs	Drug targets
Breast Neoplasms	145	64	70
Carcinoma, Non-Small-Cell Lung	98	58	84
Leukemia, Myeloid, Acute	76	43	83
Lymphoma, Non-Hodgkin	58	42	45
Multiple Myeloma	49	33	44
Leukemia, Lymphocytic, Acute	48	38	45
Colorectal Neoplasms	47	38	86
Ovarian Neoplasms	33	25	36
Stomach Neoplasms	29	26	37
Pancreatic Neoplasms	25	31	69
Prostatic Neoplasms	25	29	68
Hodgkin Disease	24	28	36
Leukemia, Lymphocytic, Chronic	17	16	29
Melanoma	17	23	30
Esophageal Neoplasms	17	18	25

**Figure 2:**
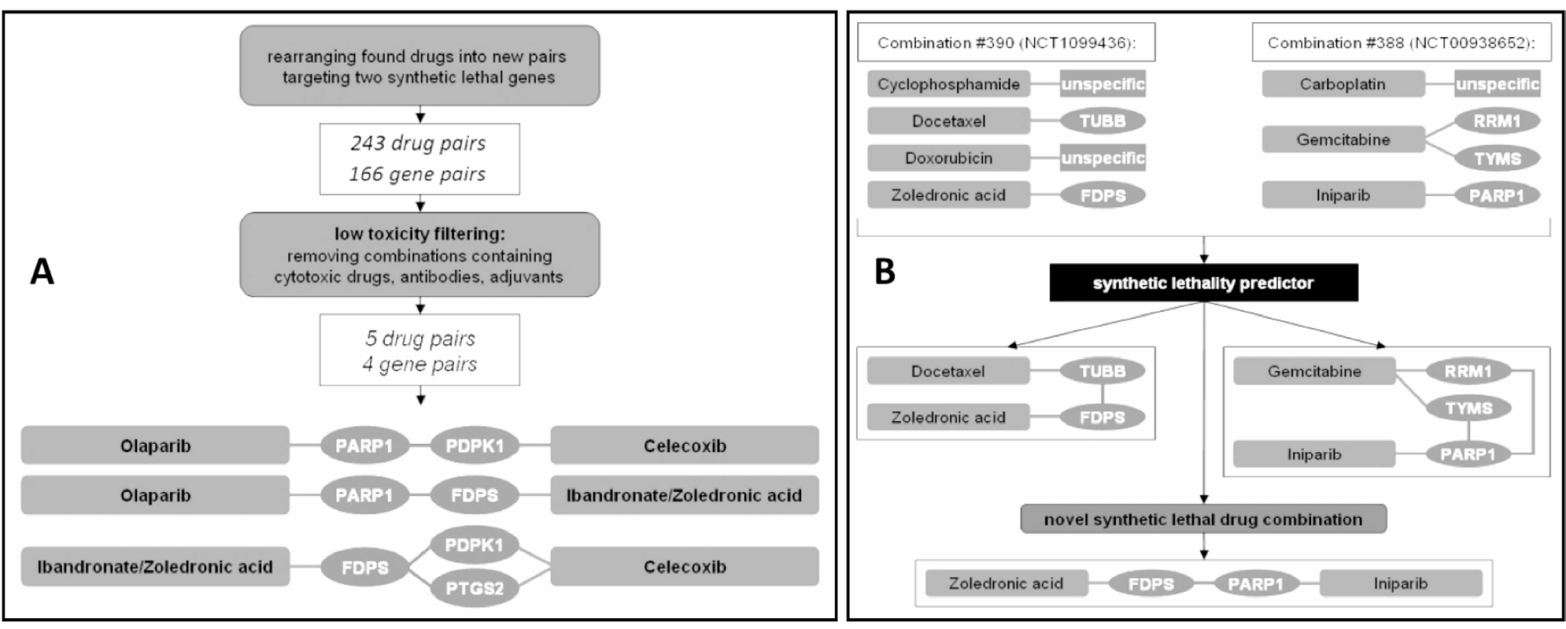
Predicting new drug combinations based on current breast cancer therapy regimens (**A**) Of 243 drug pairs covering 166 gene pairs, only 5 drug pairs were found to be non-cytostatic, low-toxicity profile drugs and were further selected for *in vitro* analysis. (**B**) In this example, combination #390 contained the lethal pair docetaxel and zoledronic acid (targeting TUBB and FDPS), while combination #388 held iniparib and gemcitabine (targeting PARP1 and both RRM1 and TYMS; only predicted drug targets relevant for this figure are depicted for combinations #388 and #390). Although not analyzed together in either trial, the combination of iniparib and zoledronic acid was suggested to target a synthetic lethal pair. A list of each drug and gene pair can be found in a Supplementary Dataset 1.

The drugs were then rearranged to form novel combinations, targeting synthetic lethal gene pairs as suggested by our *in silico* predictor based on a machine-learning algorithm. After filtering the resulting list for low toxicity combinations, the drug pairs celecoxib/zoledronic acid (ZOL/CEL) and olaparib/zoledronic acid (ZOL/OLA) were selected for further *in vitro* evaluation (Figure 2).

### Predicted synthetic lethality in breast cancer confirms highly efficient drug combinations already used in clinical routine

**Figure 3:**
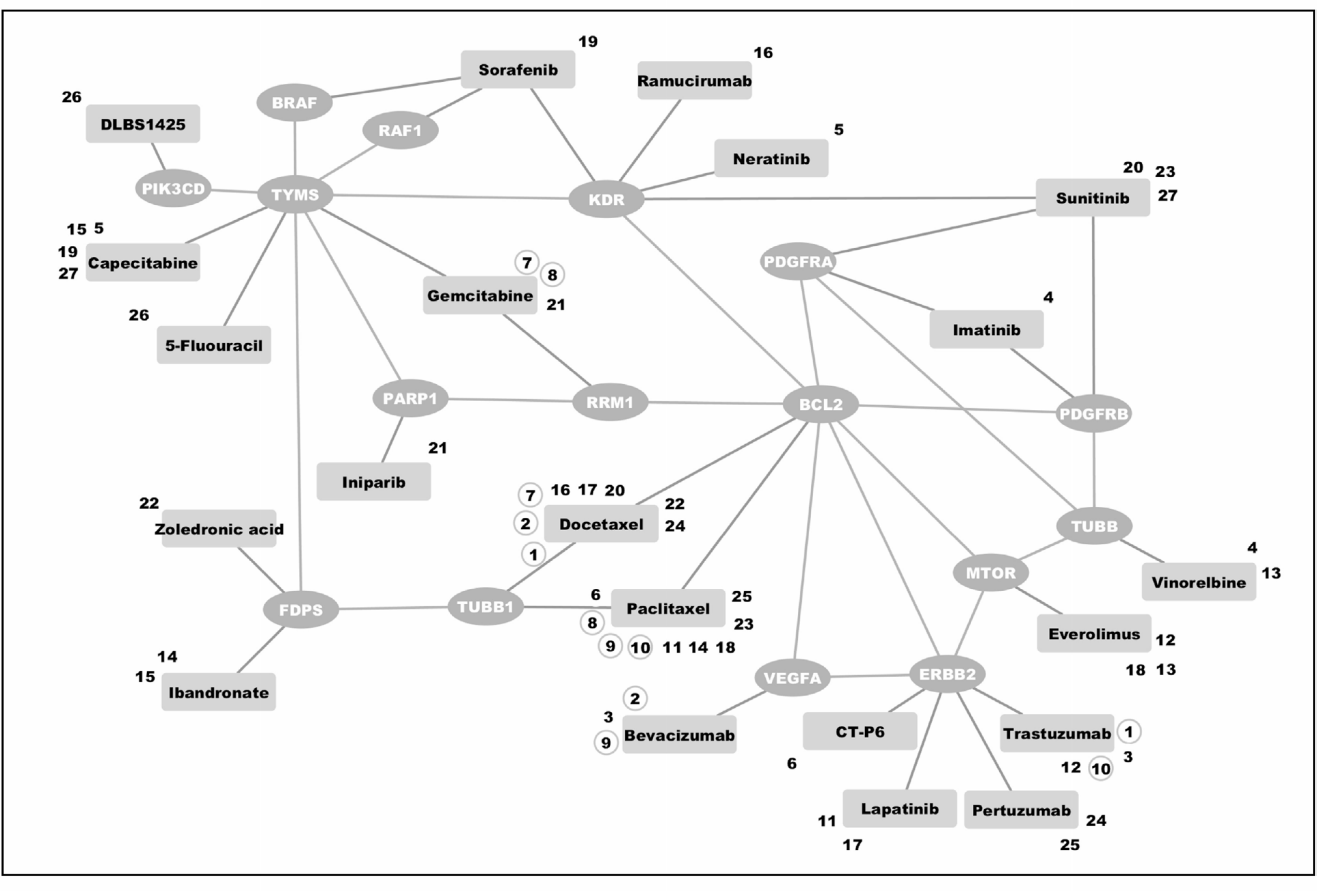
Predicted synthetic lethal interactions among drugs currently used in clinical practice or clinical trials for breast cancer Drugs found were grouped into pairs covering synthetic lethal gene pairs according to our *in silico* prediction. Zoledronic acid and docetaxel (as indicated by combination number 22), for instance, may work synergistically by targeting FDPS and TUBB1. Combination numbers in circles link drugs used as combination treatment in clinical practice. A detailed list of drugs and their assigned targets is listed in Supplementary Table 1.

Among drugs already used in clinical practice, the predictor identified six drug pairs potentially targeting gene pairs in a synthetic lethal manner. These six combinations consisted of bevacizumab, docetaxel, gemcitabine, paclitaxel, and trastuzumab (Table 2 and Figure 3).

**Table 2:** Breast cancer drug combinations used in clinical practice with their supposed synthetic lethal targets

Drug combination		Target combination	
Drug A	Drug B	Target A	Target B
Bevacizumab	Docetaxel	VEGFA	BCL2
Bevacizumab	Paclitaxel	VEGFA	BCL2
Gemcitabine	Docetaxel	RRM1	BCL2
Gemcitabine	Paclitaxel	RRM1	BCL2
Trastuzumab	Docetaxel	ERBB2	BCL2
Trastuzumab	Paclitaxel	ERBB2	BCL2

Abbreviations: VEGFA: vascular endothelial factor a; BCL2: b-cell lymphoma 2; RRM1: ribonucleotide reductase m1; ERBB22 or Her2, EGFR family receptor 2.

### Predicted drug combinations significantly reduce viability of breast cancer cells *in vitro*

**Figure 4:**
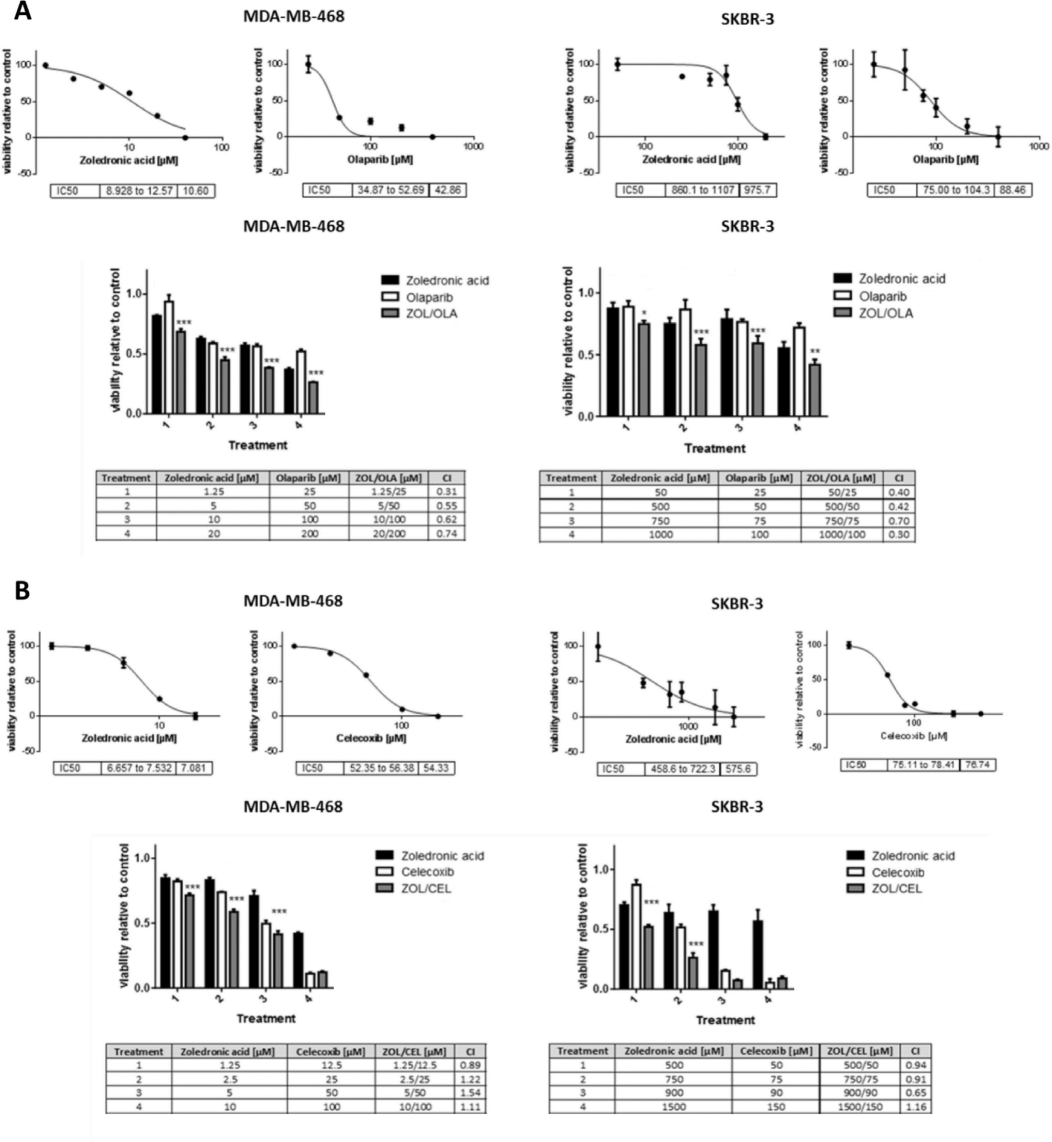
Viability assays of breast cancer cells treated with predicted synthetic lethal drug pairs Dose-response curves and IC50 values of single agent treatments (upper panels) and viability assay results of combinational treatments (lower panels) of zoledronic acid and olaparib (**A**, ZOL/OLA) and zoledronic acid and celecoxib (**B**, ZOL/CEL) in indicated cell lines at increasing drug concentrations. Cells were treated for 48 (A) or 72 hours (B). Combinational treatments with both ZOL/OLA and ZOL/CEL significantly reduced viability in comparison to either single agent treatment. Combinational indices (CI) below 1 (bottom tables) indicate synergism. Bars represent normalized mean values and error bars indicate standard error of the mean (SEM) of three technical replicates. Statistical significance of effects of synergistic treatment compared to single treatments was determined as described in “Materials and Methods” and is indicated by asterisks using adjusted *p*-values (^*^p < 0.05, ^**^p < 0.01 and ^***^p < 0.001). All experiments were performed at least three times, a representative figure is shown.

When treated with the drug combinations suggested by our synthetic lethality predictor, breast cancer cell lines MDA-MB-468 and SKBR-3 exhibited decreased viability in a significant and synergistic manner. Both drug combinations showed a significant reduction in cell viability compared to single agent treatment in both cell lines (Figure 4). Surprisingly, the observed synergy was most pronounced at lowest concentrations used. Most noteably, the combination of olaparib and zoledronic acid showed a favourable combinational index at all concentrations and in both cancer cell lines studied.

In MCF12A cells derived from benign mammary epithelium, on the other hand, combination treatment with either ZOL/CEL or ZOL/OLA did not cause synergistic declines in cell viability, indicating cancer-specificity of the effects observed (Supplementary Figure 4C).

**Figure 5:**
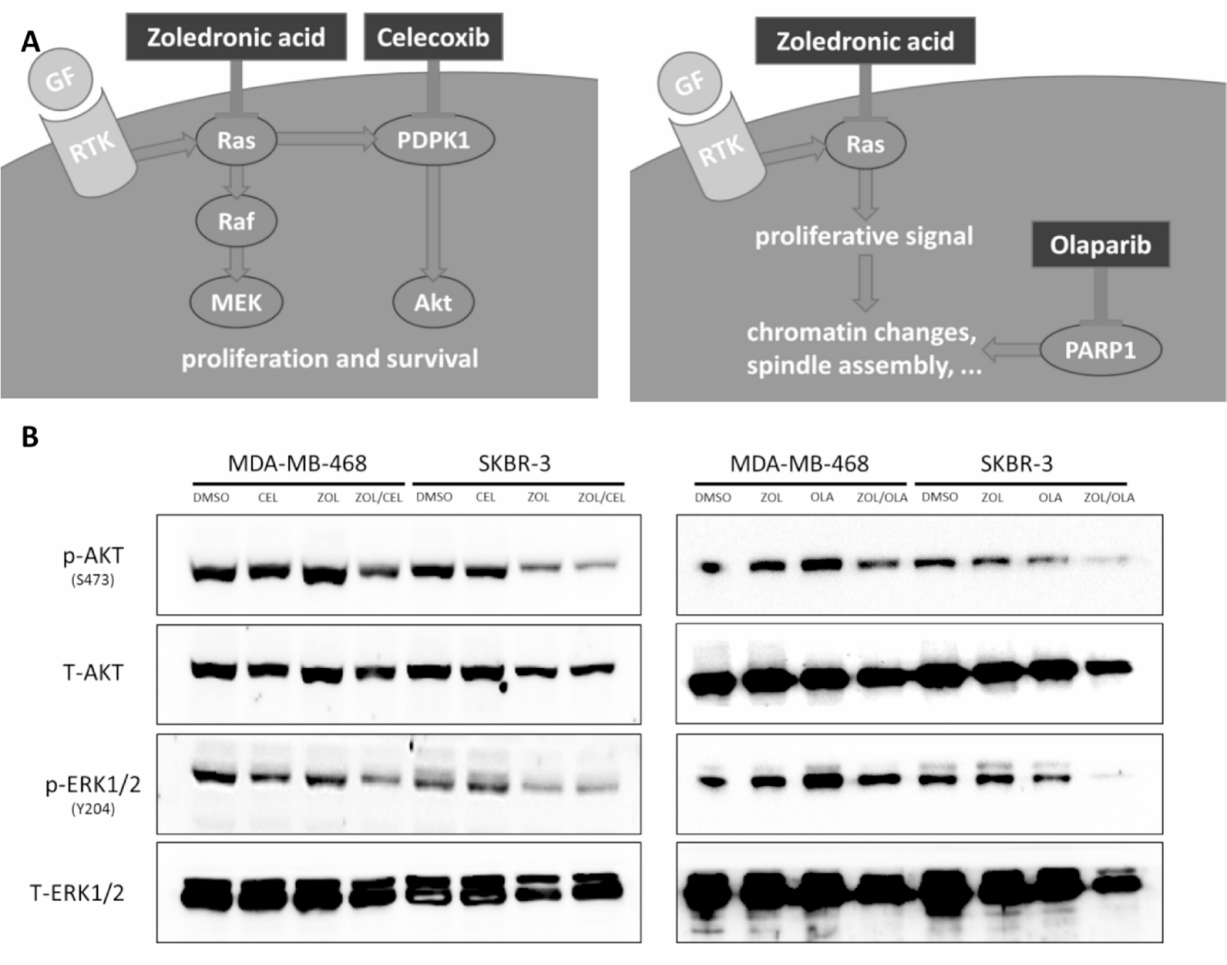
Suggested mechanism of drug interactions found (**A**) *In silico* prediction of synthetic lethality using a yeast-based screen was found for the two drug pairs of zoledronic acid and celcoxib (left) as well as zoledronic acid and olaparib (right). Zoledronic acid inhibits Ras activation by interfering with prenylation. Celecoxib blocks phosphoinositide-dependent kinase-1 (PDPK1), causing disruption of signaling of the Akt pathway. PARP inhibitors disrupt the coordination of chromatin changes and spindle assembly, leading to hindered cell division when combined with zoledronic acid, simultaneously blocking anti-apoptotic signals via Ras inhibition. (**B**) Western Blot analyses showing disruption of Akt and Erk signaling upon combination treatment of ZOL/CEL (left) and ZOL/OLA (right) in SKBR-3 and MDA-MB-468 cells treated at their respective IC50s for 48 hours. Representative blot of three independent experiments is shown.

Compatible with our findings on cell viability, immunoblotting analyses substantiate the suggested disruption of antiapoptotic and proliferative signaling through Akt and Erk in breast cancer cells upon treatment with ZOL/CEL and ZOL/OLA (Figure 5B). Further, reductions in cell viability observed were shown to be caused in part by induction of apoptosis using AnnexinV/7-AAD stainings in both MDA-MB-468 and SKBR-3 cells (Supplementary Figure 3).

### Triple-negative breast cancer cells are highly susceptible to zoledronic acid treatment

We observed a more than 100-fold difference of zoledronic acid-related cytotoxicity between the two cell lines studied, which lasted in repetition (Figure 4A). The MDA-MB-468 cell line derives from triple-negative breast cancer (TNBC) and strongly responded to zoledronic acid treatment, while Her2/neu overexpressing SKBR-3 cells did not respond in a similar manner. We were able to further confirm TNBC sensitivity towards zoledronic acid treatment using the HTB-26 cell line (Supplementary Figure 4A, 4B). To our knowledge, the observed effect of zoledronic acid on TNBC cell viability has not been described before in such significance.

## DISCUSSION

In our work, we identified novel and targetable synthetic lethality partner genes in breast cancer using an *in silico* approach based on gene interactions in yeast and known molecular drug targets for human cancer. The advantage of such cross-species methodologies, which have led to discovery of various synthetic lethal partner genes [15, 16], lies in their reliability as well as cost-efficiency, when compared to large-scale genetic knockout or knockdown experiments [13, 17–19]. However, due to evolutionary conservation, the found gene interactions might cause higher side effects when targeted, which could be seen as a disadvantage compared to large-computational cancer-specific efforts [20]. Because of this excess toxicity concern, this study focused on drug combinations comprising of relatively non-cytotoxic treatment partners, such as celecoxib and zoledronic acid, and evaluated their biological activity when tested *in vitro*.

Zoledronic acid is known to inhibit bone degradation in osteoporosis and to prevent bone metastasis in breast cancer patients [21, 22]. It has also been shown to prolong survival in premenopausal women with endocrine-responsive early breast cancer [23, 24]. Zoledronic acid belongs to the chemical group of nitrogenous bisphosphonates. One trait to this class of molecules is the blockade of the mevalonate pathway, which has been shown to be essential for prenylation and hence anchoring and activation of Ras at the cell membrane [25, 26]. Consistent with this mechanism, nitrogen-containing bisphosphonates were shown to exhibit anti-tumor activity *in vitro* by inducing apoptosis or blocking invasion and spreading [27, 28]. Other targets of zoledronic acid according to the DrugBank database include geranylgeranyl pyrophosphate synthase (GGPS1) and hydroxylapatite [29, 30]. Zoledronic acid was further shown to reverse epithelial-to-mesenchymal transition (EMT) and inhibit self-renewal in TNBC [31]. Differential activity of zoledronic acid in the two cell lines studied may be explained through pathway addiction to EGFR and hence Ras activity in triple-negative breast cancer [32]. The potential beneficial effects are also reflected in a recent meta-analysis showing a trend for improved patient outcomes in women suffering from TNBC who had been treated with zoledronic acid [33].

Celecoxib targets cyclooxygenase 2 (COX-2), but – according to the DrugBank database used for creation our model – further affects six other targets such as 3-phosphoinositide-dependent kinase-1 (PDPK1), a known activator of Akt [34–36]. Besides, cytostatic effects of celecoxib combined with sorafenib have been observed *in vitro* in various cancer cell lines [37, 38]. Additionally, celecoxib was shown to benefit patients with familiar adenomatous polyposis (FAP) by significantly reducing numbers of colorectal polyps [39].

Olaparib is a PARP1 inhibitor, a drug class whose discovery and use is based on synthetic lethality [5, 6, 40–42]. PARP1 detects and binds to DNA lesions, then produces poly(ADP-ribose) (PAR) chains, which function as a scaffold for the cellular DNA repair machinery [41]. Its inhibition has been shown to either cause deficient repair of single-strand breaks or to trap PARP at the damage site, thus creating an obstacle during the creation of the replication fork [7, 9, 43].

We reason that the synthetic lethal synergisms predicted by our yeast-based *in silico* approach and tested *in vitro*, are caused by simultaneous disruption of two interdependent pathways: Zoledronic acid interferes with the activation of Ras by inhibiting its prenylation while celecoxib blocks activation of Akt by inhibiting PDPK1. As generally accepted in the scientific community, both Ras and Akt pose crucial proliferative and antiapoptotic stimuli that cause cell death when successfully antagonized (Figure 5A). Olaparib, blocking nuclear changes following a proliferative signal, might cause cellular vulnerability in case of disruption of a second mitogenic signaling effector, such as Ras. In addition, a potential relationship between Ras and PARP was suggested by Liu and colleagues, who found PARylation of H-Ras to be important for stabilization of H-Ras and normal cell cycle progression [44].

Both cell lines used in our study are known to express wild-type BRCA1, which explains the moderate response to olaparib monotherapy as observed in our experiments. Given the fact that neither BRCA1/2 nor PARP1 exist as homologues in yeast [45, 46], we argue that our *in silico* prediction points not to vulnerability of DNA repair pathways and PARP, but rather to a not yet fully understood mechanism of PARP in regulating cellular growth and mitosis, ultimately targeting proliferative pathways such as PI3K and ERK by involvement in nuclear changes during mitosis, like loosening of chromatin or mitotic spindle assembly [47, 48]. The surprising combinatory effect seen when combined with zoledronic acid should hence encourage the community to evaluate olaparib use in indications aside from DNA-repair deficient tumors.

The synthetic lethal drug combinations identified by our predictor among drugs used in clinical practice all centered about interactions with BCL2 (Table 2). Docetaxel and paclitaxel, primarily targeting tubulin, induce off-target Bcl-2 inhibition by causing permanent Bcl-2 phosphorylation during mitotic arrest [49, 50].

Vascular Endothelial Growth Factor (VEGF) has been shown to induce Bcl-2 expression and anti-apoptotic signaling [51–53]. Her2 overexpression has also been found to cause Bcl-2 overexpression and consequent resistance to apoptosis [54, 55]. *In vitro* experiments have demonstrated that Bcl-2 expression and stability is regulated by the M2 subunit of the ribonucleotide reductase 1 (RRM1) [56].

Taken together, the synergistic drug combinations found in clinical practice all hint at simultaneous disruptions of interdependent pathways. Among drug combinations found in clinical practice, our predictor identified 23 drug pairs as well as 17 different gene pairs targeting BCL2. The recent success of the Bcl-2 inhibitor venetoclax in lymphoid and myeloid malignancies illustrates how found combinations may be of interest for future experiments [57, 58].

Although we did not observe full lethality for cells treated within our experiments as might be expected from the concept of synthetic lethality, we propose that the significant reduction of cell viability seen was caused by activation of synthetic lethal pathways. The lack of fully lethal effects in our study may be caused by insufficient pharmacological blockade of the pharmacological targets examined. Furthermore, an effect described as “synthetic sickness”, where joint deletion of two genes would lead to a less fit but viable phenotype, might be the mammalian equivalent phenomenon to synthetic lethality in the genetically less complex yeast species [13, 59].

A potential bias inherit to the design of our study is that drug selection for combination treatments relied on one specific target as listed by DrugBank. In other words, multiple targets exist for most compounds, and the target inhibition driving the effects observed in our study cannot be distinguished with absolute certainty. As an example, dependent on the concentration used and according to the DrugBank database, celecoxib will not just inhibit PDPK1 but also six other molecular targets, amongst them the primary target COX-2 (PTGS2) [29, 30]. Although we see the experimental data presented in this article as a proof-of principle of the *in silico* drug selection approach for synthetic lethal target inhibition, our results might be confounded by such positive molecular “off-target” effects by the compounds themselves, which might only occur upon their combination *in vitro*. This concern is further augmented by the use of supraphysiological drug concentrations in our study, which are needed to observe meaningful toxicity *in vitro*. Therefore, we highlight the need for additional experiments with combined gene knockouts of the molecular targets predicted, as well as additional *in vivo* and clinical studies, to further evaluate the synthetic lethal effects found through our bioinformatical approach. Also, further studies separately investigating molecular subtypes of breast cancer using a comparable approach are dearly needed to address concerns about potential subtype specificity of our findings.

## MATERIALS AND METHODS

### Translation of synthetic lethal gene pairs into human orthologues

Genetic interactions for Saccharomyces cerevisiae were obtained from DRYGIN, a synthetic genetic array covering more than 5 million gene pair interactions [60, 61]. For identifying negative genetic interactions (i.e. synthetic lethal protein coding gene pairs), the cutoff for *p*-value and genetic interaction score were set to 0.05 and –0.08, respectively. Human orthologues of yeast genes were retrieved utilizing “roundup” [62], “oma browser” [63], “ensembl” [64], “inparanoid” [65] and “HomoloGene” [66].

### *In silico* prediction of synthetic lethality gene interactions in human breast cancer

For expanding the set of synthetic lethal interactions retrieved from yeast, a computational inference method was applied. For building a classifier allowing inference of synthetic lethal interactions beyond given orthologue information from yeast, data including KEGG and PANTHER pathway identifiers [67, 68], Gene Ontology assignment according to PIR slim [69], disease association according to NCBI Medical Subject Headings (MeSH) terms [70] and drug association according to DrugBank (target, enzyme, transporter and carrier associations) was used (https://www.drugbank.ca) [29, 30]. To parameterize synthetic lethal interactions Dice’s coefficients for annotation of endpoints, the mean of node-based graph-measures and the shortest path between synthetic lethal nodes were calculated. Subsequently, a machine learning algorithm (random forest model) was created with a training set and validated on a test set of gene interactions, as previously described by our group [71]. For detailed description of predictor generation see Supplementary Figure 1.

### Identification of new synergistic drug combinations

To create new and potentially synergistic drug combinations, data on current pharmacological cancer therapy was collected. Following this step, rearrangement of drugs into new combinations targeting at least one synthetic lethal gene pair based on *in silico* prediction was performed. Treatment combinations from clinical practice and phase III/IV clinical trial information were obtained from clinical guidelines and “http://clinicaltrials.gov” [72] in August 2012 by searching for the term “cancer”. Indications were unified according to MeSH [70]. Drugs from eligible combinations were listed by indication and assigned to their targets using DrugBank. A schematic overview of this process can be seen in Supplementary Figure 2.

### Cell culture and reagents

Human breast cancer cell lines MDA-MB-468 (ATCC^®^ HTB 132™) and SKBR-3 (ATCC^®^ HTB-30™) as well as the human benign mammary epithelial cell line MCF12A (ATCC^®^ CRL-10782™) were purchased from the American Type Culture Collection (ATCC) and maintained at 37° C in a humidified atmosphere with 5% CO_2_. ATCC^®^ HTB-26™ (MDA-MB-231) cells were kindly provided by Walter Berger, Institute of Cancer Research, Medical University of Vienna. Cells were cultivated in either Dulbecco‘s Modified Eagle Medium or McCoy‘s 5a Medium Modified (only SKBR-3), supplemented with 10% fetal bovine serum and 50 units/ml penicillin G, and 50 µg/ml streptomycin sulfate. Celecoxib (CEL) and zoledronic acid (ZOL) were purchased from Sigma-Aldrich (Sigma, St. Louis, MO, USA). Olaparib (OLA) was provided by AstraZeneca (AstraZeneca, London, United Kingdom). Drugs were diluted in DMSO (CEL and OLA) or PBS (ZOL) respectively.

### Viability assays

IC_50_ values were determined by performing dose-response curves by treating cells in a dose escalating manner. Cells were treated in a dose escalating manner. 2500 cells were plated in 50 µl of culture medium in 96-well plates overnight. The next day, 50 µl of medium containing the double amount of the desired concentration was added and incubated. Cell survival was measured at time points up to 72 hours by using CellTiter-Blue^®^ (Promega, Madison, WI, USA) according to the manufacturers’ protocol. Treatments were conducted in triplicates, averaged, and standardized to control (DMSO treatment). All statistical computations were performed using Prism 6 (GraphPad Software, Inc., USA). Multiple group analysis was performed using two-way analysis of variance (ANOVA) and Dunnetts’ multiple comparison test as a post-hoc test. Synergy was quantified utilizing CompuSyn and by calculating the combinational index [73].

### Immunoblotting

200.000 cells were cultivated in a 12-well format and treated with IC50 concentrations for single or combinational treatment for 48 hours. Proteins were isolated using RIPA lysis buffer supplemented with cOmplete Protease Inhibitor Cocktail Tablets (Roche Diagnostics, Mannheim, Germany) and PhosSTOP Phosphatase Inhibitor Cocktail Tablets (Roche Diagnostics). Protein concentration was measured using Bradford reagent (Sigma-Aldrich, St. Louis, Missouri, USA). 30 µg of protein was loaded and separated on a 10% polyacrylamid gel in a Tris-Glycin-SDS buffer. Proteins were then blotted onto nitrocellulose membranes using Turboblot (Biorad, Hercules, California, USA). Membranes were blocked in 5% bovine serum albumin (Sigma-Aldrich) diluted in TBS-T and incubated with primary antibodies overnight at 4° C. Primary antibodies used were P-AKT Ser473 (Cell Signaling, #4060), T-AKT (Cell Signaling, #), P-ERK1/2 Tyr204 (Santa Cruz, sc-101761), P-ERK1/2 Thr202/Tyr204 (Cell Signaling, #9101), T-ERK 1/2 (Santa Cruz, sc-93/sc-154). Visualization was performed by using horseradish peroxidase (HRP)-conjugated secondary antibodies (Santa Cruz Laboratories, Dallas, Texas, USA) and the enhanced chemiluminescence detection system (Biorad) on a ChemiDoc XRS+ Imaging device (Biorad).

### Annexin/7-AAD stainings

200.000 cells were seeded into wells of 12-well plates and left to adhere overnight. Cells were treated at the IC50 of each compound. At indicated time points, cells were then trypsinized and stained with AnnexinV-APC (BD Bioscience, 550474) and 7-AAD (ebioscience, 00-6993-50) according to the manufacturers protocol. Cell fluorescence was captured using a FACS Canto II device (BD Bioscience) and analyzed using FlowJo software (FlowJo LLC, V10).

## SUPPLEMENTARY MATERIALS FIGURES AND TABLES





## Abbreviations

PARP: Poly (ADP-ribose) polymerase
BRCA1/2: Breast Cancer susceptibility gene 1/2
SSR: Single strand repair
HDR: Homology directed repair
RNA: Ribonucleic acid
CRISPR: Clustered regularly interspaced short palindromic repeats
CAS: CRISPR associated
TNBC: Triple Negative Breast Cancer
EMT: Epithelial-Mesenchymal transition
EGFR: Epidermal Growth Factor Receptor
COX-2: Cyclooxygenase 2
PDPK1: Phosphoinositide-dependent kinase-1
FAP: Familial adenomatous polyposis
PAR: Poly(ADP-ribose)
DNA: Deoxyribonucleic acid
VEGF: Vascular endothelial growth factor
RRM1: Ribonucleotide reductase 1
IC50: Half maximal inhibitory concentration
ATCC: American Type Culture Collection
CO2: Carbon dioxide
DMSO: Dimethyl sulfoxide
7-AAD: 7-Aminoactinomycin D
