# Supplementary material for: Synthetic lethal combinations of low-toxicity drugs for breast cancer identified in silico by genetic screens in yeast
Source: Oncotarget. 2018 Nov 20;9(91):36379–91. doi: 10.18632/oncotarget.26372 (PMC6284748; doi:10.18632/oncotarget.26372)
Supplement: Supplementary file 1 [file oncotarget-09-36379-s001.pdf]

Synthetic lethal combinations of low-toxicity drugs for breast cancer identified *in silico* by genetic screens in yeast

SUPPLEMENTARY MATERIALS

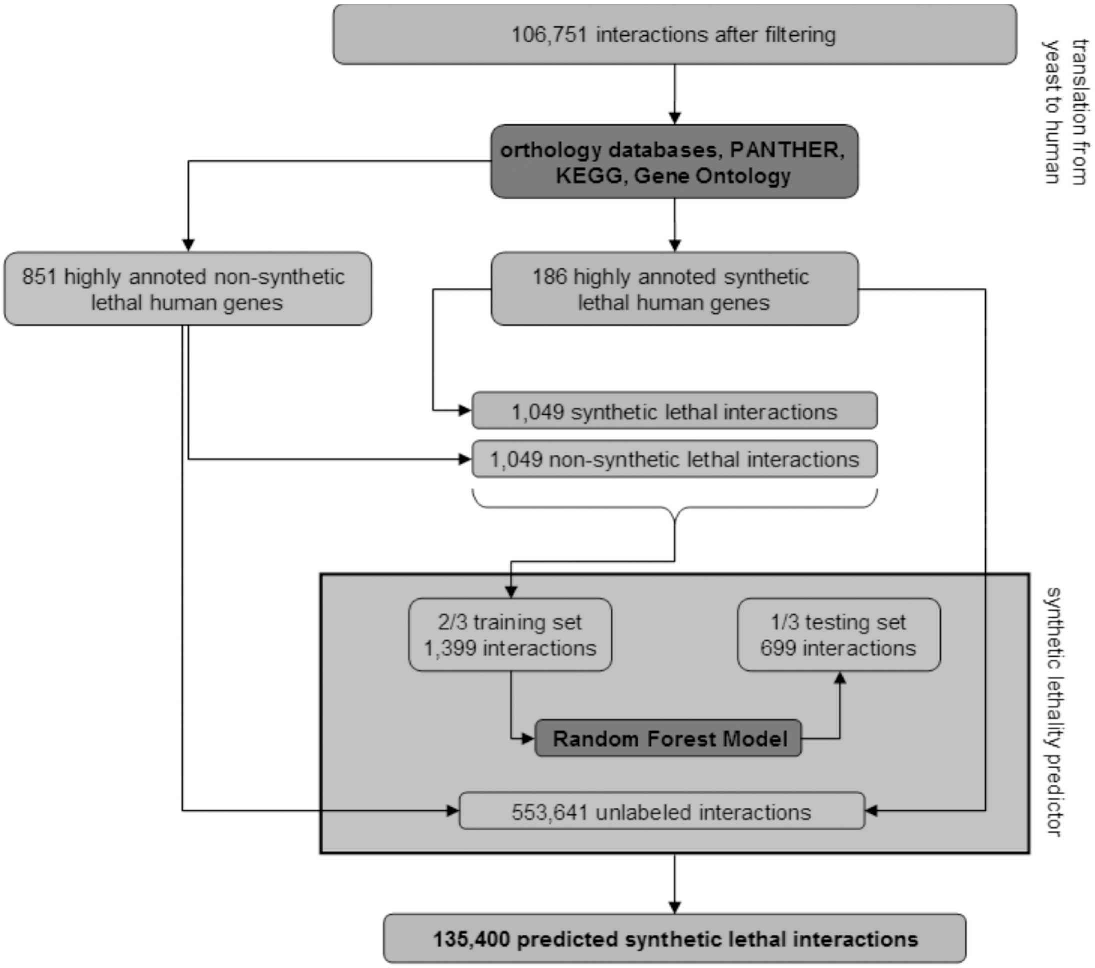

Supplementary Figure 1: Creation of a synthetic lethality predictor. After filtering data publicly available in the DRYGIN database for negative interactions, 106,751 synthetic lethal interactions in yeast remained for further analysis. Translation of found genes into human orthologues and adding data regarding gene pathway, function and drug association formed the foundation for the synthetic lethality predictor. 186 human genes derived from synthetic lethal genes in yeast met this high level of annotation. These genes formed 1,049 potential synthetic lethal interactions in men. From human genes meeting the same standard of annotation, but not participating in synthetic lethality, another 1,049 interactions were taken at random, to create a dataset of equal synthetic lethal and non-lethal gene interactions. Based on a random forest model the predictor learned to differentiate between gene pairs originating from synthetic lethality and random pairs and was later verified on a test set. By analyzing 553,641 unlabeled gene interactions, a total of 135,400 synthetic lethal gene pairs was predicted.

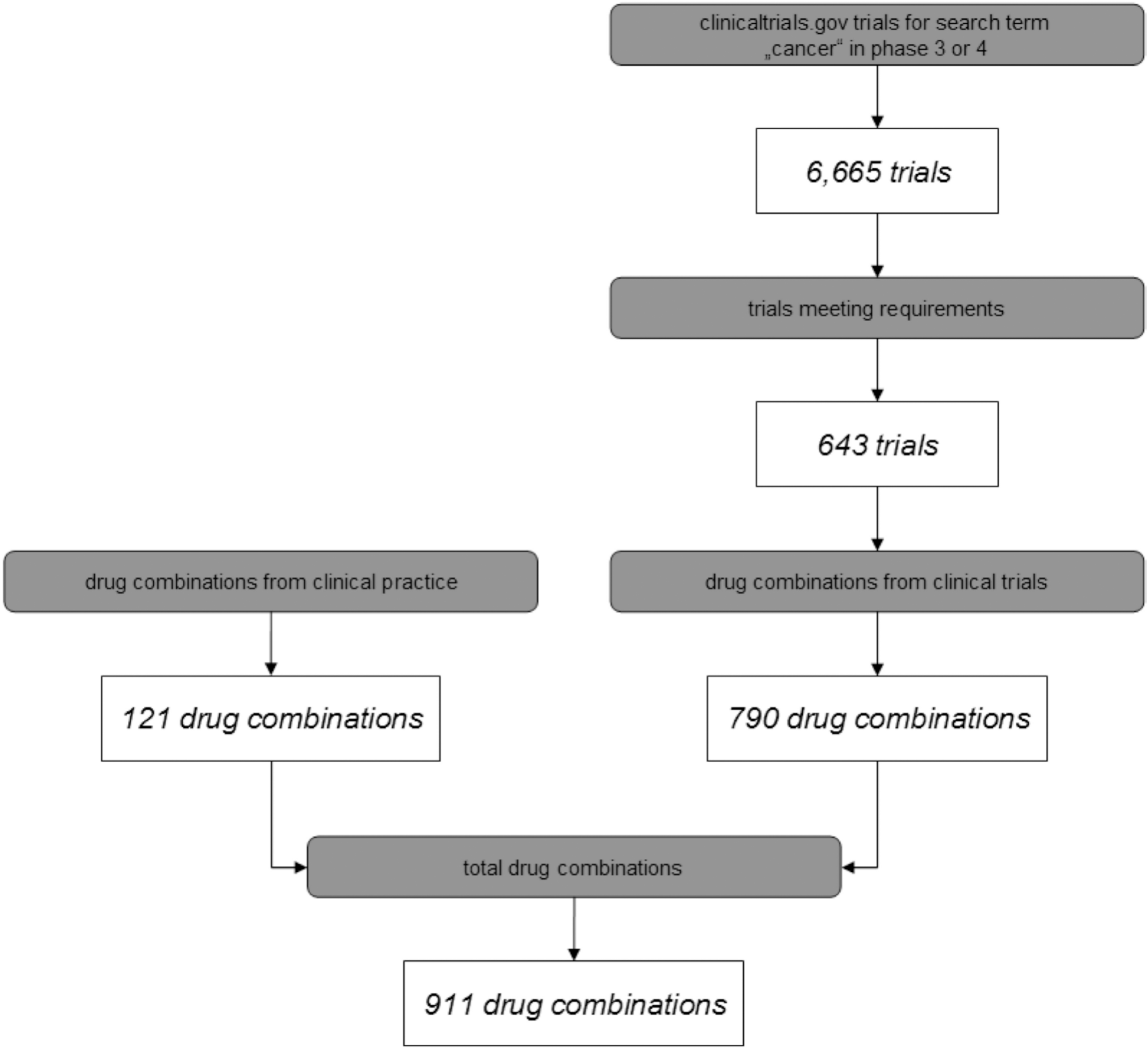

Supplementary Figure 2: Acquisition of data on current cancer therapy. 6,665 trials were obtained from clinicaltrials.gov by searching for "cancer" and limiting results for phase III and IV. 643 trials met the requirements and contained 790 different drug combinations. Together with 121 drugs from clinical practice, 911 unique drug combinations were found (see Supplementary Dataset 1).

SKBR3

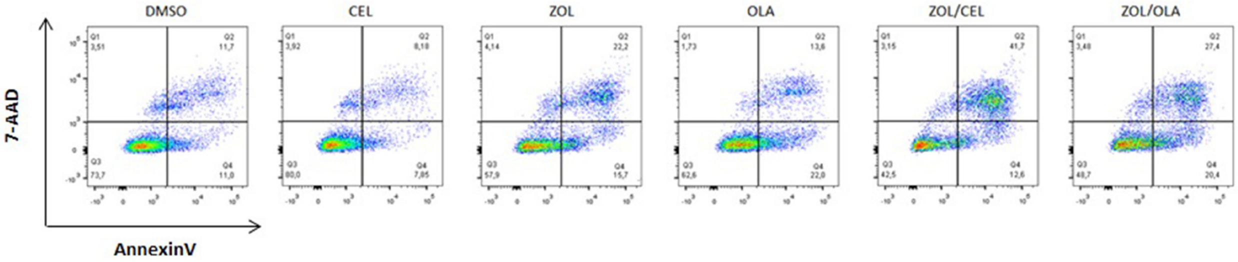

**Q1**	3,51	3,92	4,14	1,73	3,15	3,48
**Q2**	11,7	8,18	22,2	13,6	41,7	27,4
**Q3**	73,7	80,0	57,9	62,6	42,5	48,7
**Q4**	11,0	7,85	15,7	22,0	12,6	20,4
**Q2+Q4**	22,7	16,03	37,9	35,6	54,3	47,8

MDA-MB-468

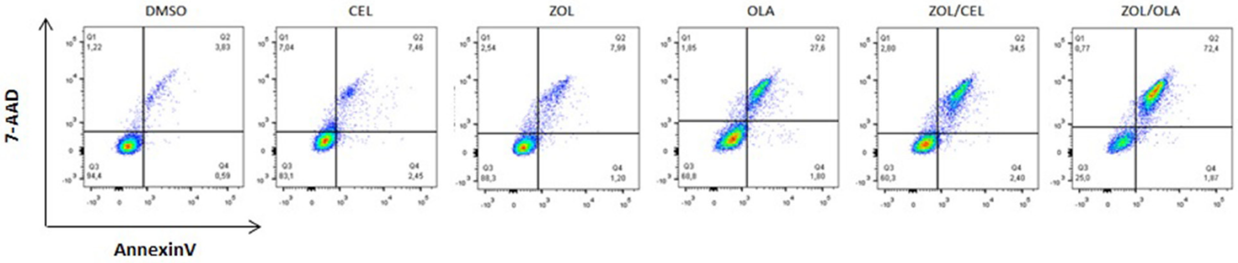

**Q1**	1,22	7,04	2,54	1,85	2,80	0,77
**Q2**	3,83	7,46	7,99	27,6	34,5	72,4
**Q3**	94,4	83,1	88,3	68,8	60,3	25,0
**Q4**	0,59	2,45	1,20	1,80	2,40	1,87
**Q2+Q4**	4,42	9,91	9,19	29,4	36,9	74,27

Supplementary Figure 3: AnnexinV/7-AAD stainings of SKBR-3 (upper panel) and MDA-MB-468 (lower panel) cells treated with celecoxib (CEL), zoledronic acid (ZOL), olaparib (OLA) or drug combinations (ZOL/CEL and ZOL/OLA). SKBR-3 and MDA-MB-468 cells were treated for 48 (SKBR-3) or 72 (MDA-MB-468) hours at empirically established IC50 values. The table illustrates increases in early (Q2) and late (Q4) apoptotic cells upon treatment (percent of total cells). Each experiment was repeated three times. Representative results of 10.000 analyzed cells are shown.

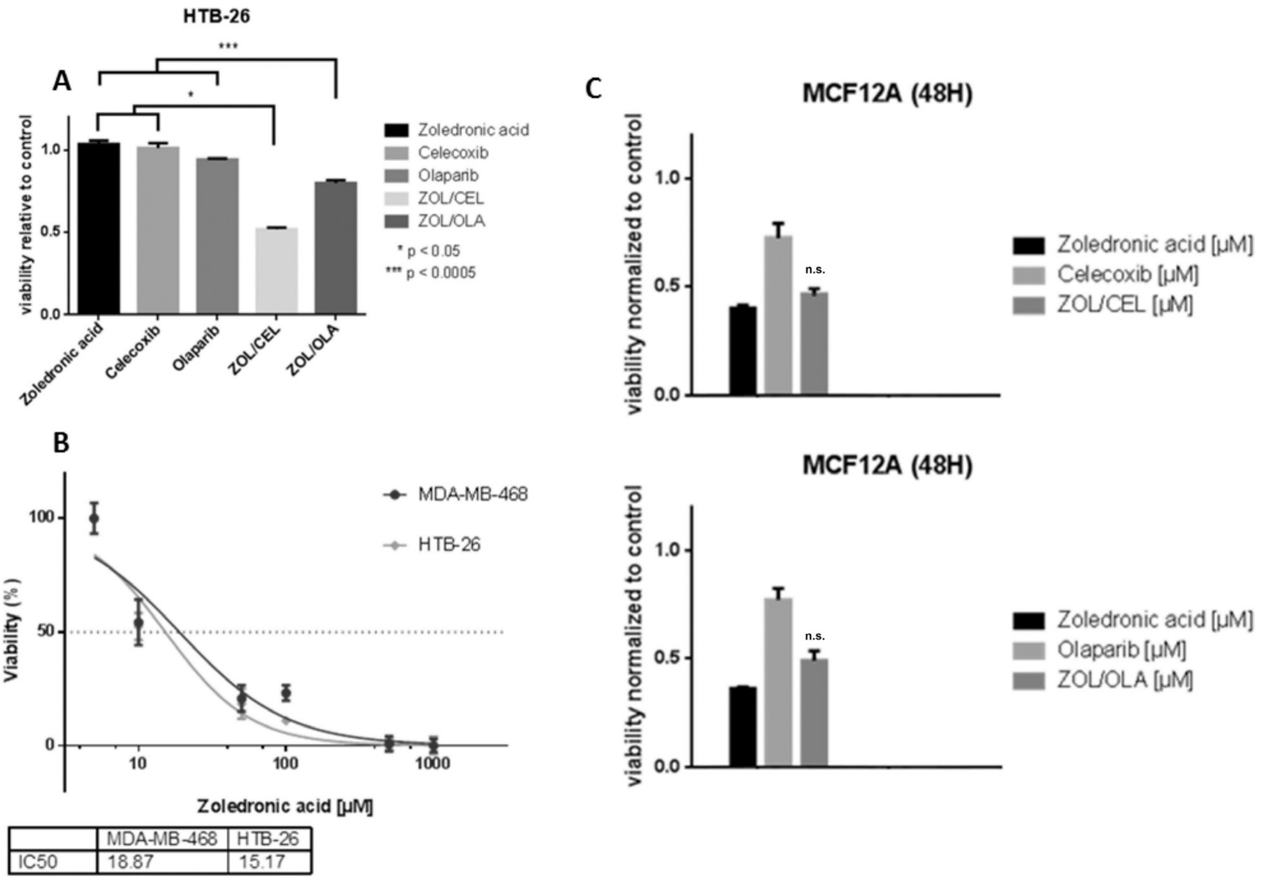

Supplementary Figure 4: Viability assays. (**A**) Synergistic effects of ZOL/CEL and ZOL/OLA on cell viability of the triple negative cell line HTB-26 treated with low drug concentrations. A third of the previously determined IC50 of each compound was used (zoledronic acid 5 µM, celecoxib 37.5 µM, olaparib 50 µM) and cells were treated for 48 hours. (**B**) Both triple negative cell lines HTB-26 and MDA-MB-468 exhibit low IC50 values upon 48 hours of treatment with zoledronic acid. (**C**) MCF12A cells derived from benign mammary tissue do not exhibit synergism when treated with ZOL/CEL (upper figure) or ZOL/OLA (lower figure) for 48 hours at previously determined IC50s (zoledronic acid 20 µM, celecoxib 150 µM, olaparib 200 µM). One representative result of at least two independent experiments with three technical replicates is shown for Figures S4A-C. Asterisks indicate statistical significance (n.s. - not significant).

Supplementary Table 1: Breast cancer drug combinations used in clinical practice and clinical trials - as depicted in Figure 3 - with their predicted synthetic lethal targets

Drug combination		Target combination	
**Drug A**	**Drug B**	**Target A**	**Target B**
Bevacizumab	Paclitaxel	VEGFA	BCL2
Bevacizumab	Docetaxel	VEGFA	BCL2
Bevacizumab	Paclitaxel	VEGFA	BCL2
Bevacizumab	Docetaxel	VEGFA	BCL2
Bevacizumab	Docetaxel	VEGFA	BCL2
Bevacizumab	Docetaxel	VEGFA	BCL2
Bevacizumab	Docetaxel	VEGFA	BCL2
Bevacizumab	Paclitaxel	VEGFA	BCL2
Bevacizumab	Docetaxel	VEGFA	BCL2
Bevacizumab	Trastuzumab	VEGFA	ERBB2
Bevacizumab	Docetaxel	VEGFA	BCL2
Bevacizumab	Trastuzumab	VEGFA	ERBB2
Bevacizumab	Paclitaxel	VEGFA	BCL2
Bevacizumab	Trastuzumab	VEGFA	ERBB2
Bevacizumab	Docetaxel	VEGFA	BCL2
Bevacizumab	Docetaxel	VEGFA	BCL2
Bevacizumab	Paclitaxel	VEGFA	BCL2
Bevacizumab	Docetaxel	VEGFA	BCL2
Bevacizumab	Trastuzumab	VEGFA	ERBB2
CT-P6	Paclitaxel	ERBB2	BCL2
DLBS1425	5-Fluouracil	PIK3CD	TYMS
Docetaxel	Zoledronic acid	TUBB1	FDPS
Gemcitabine	Docetaxel	RRM1	BCL2
Gemcitabine	Paclitaxel	RRM1	BCL2
Gemcitabine	Paclitaxel	RRM1	BCL2
Gemcitabine	Docetaxel	RRM1	BCL2
Gemcitabine	Docetaxel	RRM1	BCL2
Gemcitabine	Paclitaxel	RRM1	BCL2
Ibandronate	Capecitabine	FDPS	TYMS
Ibandronate	Capecitabine	FDPS	TYMS
Imatinib	Vinorelbine	PDGFRB	TUBB
Imatinib	Vinorelbine	PDGFRA	TUBB
Iniparib	Gemcitabine	PARP1	RRM1
Iniparib	Gemcitabine	PARP1	TYMS
Lapatinib	Paclitaxel	ERBB2	BCL2
Lapatinib	Paclitaxel	ERBB2	BCL2
Lapatinib	Paclitaxel	ERBB2	BCL2
Lapatinib	Paclitaxel	ERBB2	BCL2
Lapatinib	Docetaxel	ERBB2	BCL2
Neratinib	Capecitabine	KDR	TYMS
Paclitaxel	Ibandronate	TUBB1	FDPS
Paclitaxel	Ibandronate	TUBB1	FDPS
Paclitaxel	Everolimus	BCL2	MTOR
Pertuzumab	Docetaxel	ERBB2	BCL2
Pertuzumab	Trastuzumab	ERBB2	EGFR
Pertuzumab	Paclitaxel	ERBB2	BCL2
Pertuzumab	Trastuzumab	ERBB2	EGFR
Ramucirumab	Docetaxel	KDR	BCL2
Sorafenib	Capecitabine	KDR	TYMS
Sorafenib	Capecitabine	RAF1	TYMS
Sorafenib	Capecitabine	BRAF	TYMS
Sunitinib	Docetaxel	PDGFRB	BCL2
Sunitinib	Docetaxel	KDR	BCL2
Sunitinib	Docetaxel	PDGFRA	BCL2
Sunitinib	Paclitaxel	PDGFRB	BCL2
Sunitinib	Paclitaxel	KDR	BCL2
Sunitinib	Paclitaxel	PDGFRA	BCL2
Sunitinib	Capecitabine	KDR	TYMS
Trastuzumab	Paclitaxel	ERBB2	BCL2
Trastuzumab	Docetaxel	ERBB2	BCL2
Trastuzumab	Docetaxel	ERBB2	BCL2
Trastuzumab	Paclitaxel	ERBB2	BCL2
Trastuzumab	Docetaxel	ERBB2	BCL2
Trastuzumab	Paclitaxel	ERBB2	BCL2
Trastuzumab	Paclitaxel	ERBB2	BCL2
Trastuzumab	Paclitaxel	ERBB2	BCL2
Trastuzumab	Docetaxel	ERBB2	BCL2
Trastuzumab	Everolimus	ERBB2	MTOR
Trastuzumab	Paclitaxel	ERBB2	BCL2
Trastuzumab	Docetaxel	ERBB2	BCL2
Trastuzumab	Docetaxel	ERBB2	BCL2
Trastuzumab	Docetaxel	ERBB2	BCL2
Trastuzumab	Docetaxel	ERBB2	BCL2
Trastuzumab	Paclitaxel	ERBB2	BCL2
Trastuzumab	Docetaxel	ERBB2	BCL2
Trastuzumab	Docetaxel	ERBB2	BCL2
Trastuzumab	Docetaxel	ERBB2	BCL2
Trastuzumab	Docetaxel	ERBB2	BCL2
Trastuzumab	Docetaxel	ERBB2	BCL2
Vinorelbine	Everolimus	TUBB	MTOR

Supplementary Dataset 1: Dataset of predicted target and gene pairs. See Supplementary_Dataset_1
